# Transactions of the Georgia State Commission on Tuberculosis

**Published:** 1905-07

**Authors:** Bernard Wolff

**Affiliations:** Permanent Secretary


### TRANSACTIONS OF THE GEORGIA STATE COMMISSION ON TUBERCULOSIS.

### PROGRAMME.

Session convenes at 9 o'clock a.m.

Remarks by the Chairman.

Election of Permanent Secretary.

Short Addresses by Doctors H. F. Harris, Atlanta, Secretary State Board of Health, Pathologist, Biologist and Bacteriologist College of Physicians and Surgeons; Henry McHatton, Macon, ex-President Medical Association of Georgia, Member of Bibb County and City of Macon Boards of Health; George Brown, Atlanta, Secretary American Anti-Tuberculosis League; T. E. Oertel, Augusta, Chairman Committee on Tuberculosis Medical Association of Georgia, Pathologist and Bacteriologist School of Medicine University of Georgia.

After the addresses the members of the Commission in Committee of the Whole will discuss and act upon the Sections of the Resolution by the Legislature authorizing the Governor to raise the Commission on Tuberculosis.

Appointment of Committees.

The Georgia State Commission on Tuberculosis was called to order by Dr. Chas. Hicks, Chairman, at 9:15, October 19, in the parlors of the Lanier Hotel, Macon, Georgia. The meeting was opened by prayer by Rev. Dr. W. N. Ainsworth, of Mulberry Street Methodist Church of Macon. Dr. T. E. Oertel, of Augusta, Chairman of the Tuberculosis Committee of the Georgia Medical Society, was requested by the Chairman to act as Temporary Secretary. The Chairman then announced that he had invited the Committee on Tuberculosis from the Georgia Medical Society to cooperate with the Commission, and that he had also requested Dr. Geo. Brown, of Atlanta, President of the Anti-Tuberculosis League of America, Dr. H. F. Harris, of Atlanta, Secretary of the State Board of Health, and Dr. H. McHatton, of Macon, a Member of the Board of Health of Bibb County and of the City of Macon, to be present and deliver addresses.

The following members of the Commission were present: Chas. Hicks, L. H. Jones, E. P. Ham, P. L. Hilsman, P. W. Fitts, L. J. Bell, Alex. Mack, H. R. Slack, Bernard Wolff, Geo. T. Alexander, S. A. Brown, Henry E. Thornton, Jefferson Davis, Thos. D. Coleman. The following members of the Committee from the

State Medical Association were in attendance: T. E. Oertel, R. R. Kime, R. H. Thompson, M. A. Clark. Drs. George Brown, H. McHatton and H. F. Harris were also present.

The Chairman then delivered his address (q. v. page 512).

The election of Permanent Secretary resulted in the unanimous election of Dr. Bernard Wolff, of Atlanta.

Dr. H. McHatton then delivered his address, the subject of it being "Is Tuberculosis a Disease of Environment Only?" (q. v. page 518.)

Dr. George Brown, of Atlanta, then addressed the Commission. He spoke without manuscript. He said that he was optimistic concerning the work of the Commission, and that he believed that the next Legislature, without much urging, would provide the funds for the necessary expenditures in the carrying on of the work. He said that at the time that there was introduced the bill on the floor of the Senate by Hon. Harvey Jordan, that there was a motion made to amend the bill so that it would receive funds with which to carry on the work, but that the motion was withdrawn by the request of those who were behind the movement, for the reason that it was the desire of the promoters of the measure to first show the people of Georgia what they proposed to do. He said that the report of the work at the next session would show what was being done, and the attitude of the physicians of Georgia.

Dr. Brown stated that he was proud of the fact that the State of Georgia was the first south of Mason and Dixon's line to take up the work, and that the profession of the State deserved all the more credit because the movement was being conducted at this time without funds and through the sacrifice and the interest of the Georgia physicians.

It was stated that from the first Governor Terrell has been the firm friend of the movement, and that they might continue to rely upon him for assistance.

Dr. H. F. Harris was the next speaker. He referred to Virchow's dictum of all cells from pre-existing cells. This dictum applies equally to infectious diseases. He called attention to the change of view which had taken place in regard to malaria and alluded to the role of the mosquito in its propagation.

From the work of Reed, Lazear and others we know that the same facts hold good in yellow fever. Tuberculosis was no exception to the rule in infectious disease. The tubercle bacillus was very susceptible to change in temperature, the temperature of the body being the most favorable for its growth. Bovine and human tuberculosis were merely modified forms of the same disease.

The diagnosis was of the greatest importance. The disease was sometimes mistaken for malaria. Care and accuracy in diagnosis should be especially emphasized. The establishment of a free laboratory for making bacteriological examinations at the State Capitol in Atlanta under the supervision of the Board of Health put a means in the hands of the physicians in the State that would greatly assist diagnosis. He hoped soon to be able to furnish free tuberculin for testing cattle. The State Board of Health was greatly crippled in its usefulness by lack of funds. Dr. Harris expressed sympathy on behalf of the State Board of Health with the anti-tuberculosis movement and its desire to cooperate fully with the Tuberculosis Commission.

Dr. T. E. Oertel, chairman of the Tuberculosis Committee, appointed by the State Medical Society, followed. He expressed the desire of his committee to cooperate with the Tuberculosis Commission. He believed that the most important step in the anti-tuberculosis movement was the education of the public to a better knowledge of the nature of the disease.

His committee was much hampered in its work by lack of money. He had appealed for contributions from the public through the daily papers. There was absolutely no response. The public was apathetic. It looked to the doctors themselves for funds to carry on the work.

He referred to tuberculosis among the negroes. The mortality among them in cities was twenty-five per cent. The percentage was probably underestimated. Many cases were not recognized and death was attributed to some other cause. The figures from Augusta hospital showed a mortality of thirty-three per cent. among negroes. In rural districts percentage was between fifteen and twenty-five. At Milledgeville asylum it was fifty per cent. The disease among negroes has increased enormously since the war. Before that time it was rare. The prevalence of the disease among negroes is a potent factor in its spread. A large number of negroes are employed as domestic servants, constituting a serious menace to the household of their employers. How to deal with negroes whose means of livelihood was taken away by enforcement of anti-tuberculosis measures was one of the phases of the problem. It is the duty of the State to provide for them, but the fulfillment of this duty would meet opposition from the public. State support of the indigent white tuberculous being difficult to secure, how much more is it in case of the negro.

The question of tuberculosis was not only medical but economic. Each life has a money value to the State and its loss means a loss to the State. Education of the laity in the matter could be undertaken in part through the schools by the distribution of literature and other means. The results in this direction will necessarily be slow, but persistent effort will see the end attained.

Laws should be enacted for the restriction of the disease and the prevention of its spread. It will be hard to secure such laws. There will be opposition and apathy as to their enforcement. Physicians, many of them, need to be admonished in the duty in regard to protection of the people against tuberculosis. Cases are sometimes not recognized; others are concealed.

Appropriation from the Legislature will not be easy to secure, but will be ultimately forthcoming if the physicians of the State are organized in the movement and exert their influence upon their representatives in the General Assembly. The Georgia Medical Association was the first to move in the matter, and the Tuberculosis Committee stands ready to assist and cooperate with the State Commission in every way.

The chairman then accorded the privileges of the floor to other members of the committee from the Society.

Dr. H. E. Thornton moved that the county papers throughout the State be asked to publish the addresses of Dr. Hicks and Dr. McHatton. Dr. S. A. Brown amended the motion to publish collected information.

Dr. Oertel mentioned that letters would be published in the local papers by members of the committee from the State Society.

Further discussion on Dr. Thornton's motion developed the opinion that the literature for distribution to the papers should be epitomized and condensed. For the purpose of editing such literature Dr. Louis H. Jones suggested the appointment of a publication committee. In this form the motion was carried.

Dr. S. A. Brown moved that a committee of five be appointed to act as a steering or executive committee, such committee to act under instructions from the Commission. Carried.

The several propositions contained in the bill creating the Commission were then taken up. The propositions were as follows: (1) The number of cases of tuberculosis in the State of Georgia. (2) The annual number of deaths from tuberculosis in the State of Georgia. (3) The number of cases contracted within the State, and the number imported. (4) The measures that are being taken in the various localities for preventing the spread of the disease. (5) Whether or not the physicians of the State are in favor of taking measures for its prevention.

In taking up the discussion of the first proposition the Chairman commented on the difficulty of obtaining statistics on the subject of tuberculosis. The disease was not reported to the health boards. Means of obtaining data must be adopted, and the best course to pursue must be outlined at this meeting. He suggested that the Chairman of this Commission and the President of the State Medical Association place themselves in correspondence with the physicians in the State for the purpose of organizing them in the work of collecting the desired statistical information.

Dr. S. A. Brown moved that each member of the Commission for congressional districts correspond with every physician in his district to secure the necessary data.

Dr. Alex. Mack amended this motion and moved that the matter be submitted to the executive committee to report later to the Commission. Carried.

The Chairman then appointed the Executive Committee—Dr. Alex. Mack, Dr. Louis H. Jones, Dr. S. A. Brown, Dr. Thos. D. Coleman, Dr. Bernard Wolff, Secretary.

The Commission then adjourned until 3 p. m.

AFTERNOON SESSION.

The Commission was called to order by the Chairman, Dr. Hicks, at 3 p. m.

Telegrams were read from Dr. O. B. Bush and Dr. J. R. Burdette, expressing regret at being unable to attend the meeting.

The Secretary then read the report of the Executive Committee.

*Resolved*, That the members of this Commission endeavor to secure statistics on each point mentioned in the bill creating this Commission, by correspondence with such physicians in their district as deemed best by the members, or by any other means, and that they report the result to the Secretary of this Commission by April 1, 1905.

That the sum of $5 be paid at once, or as soon as possible, to the secretary by each member to defray the expenses of postage, stenographic work, etc.

That THE ATLANTA JOURNAL-RECORD OF MEDICINE be declared the official organ of this Commission.

On motion, the report of the Executive Committee was unanimously adopted.

Dr. Coleman moved that the office of Secretary and Treasurer be combined. Carried.

The Chairman recommended that the district reports be sent in monthly, not later than the 20th of each month, for publication in the official organ of the Commission. He also recommended the work entitled "A Handbook on the Prevention of Tuberculosis," published by the Charity Organization Society, 105 E. Twenty-second street, New York, and sold at $1.19 including postage, and advised that each member of the Commission procure a copy of it.

Col. G. C. Matthews, of the *Macon Telegraph*, tendered the use of the wire service of his paper to the Commission, for which courtesy a vote of thanks was extended to him.

The Secretary was instructed to notify absent members of the Commission of the fund to be created by subscription.

It was decided that the next meeting of the Commission be held at Atlanta in April, 1905, just prior to the meeting of the State Medical Association, when the data collected will be epitomized and a formal report made, to be presented to the Legislature at its next session in June, 1905.

On motion, the Commission adjourned its session.

BERNARD WOLFF, M.D.,
Permanent Secretary.

SECRETARY'S REPORT GEORGIA STATE COMMISSION ON TUBERCULOSIS.

